# Septum Pellucidum Chronic Encapsulated Hematoma With Osseous Metaplasia Mimicking Recurrent Astrocytoma and Shunt-Related Foreign Body Granuloma

**DOI:** 10.7759/cureus.9839

**Published:** 2020-08-18

**Authors:** Fabio Roberti, Julie Bell

**Affiliations:** 1 Neurosurgery, Cleveland Clinic Indian River Hospital, Vero Beach, USA; 2 Neurosurgery, George Washington University School of Medicine and Health Sciences, Washington, USA; 3 Pathology, Cleveland Clinic Indian River Hospital, Vero Beach, USA

**Keywords:** chronic encapsulated hematoma, foreign body granuloma, septum pellucidum

## Abstract

We present a very rare case of chronic encapsulated intracerebral hematoma involving the septum pellucidum and the foramen of Monro that by location radiological appearance, and clinical history was mimicking a recurrent astrocytoma or a shunt-related foreign body granuloma.

A young adult underwent the resection of a juvenile pilocytic astrocytoma as a child, and with a mass encasing the tip of an old non-functioning ventricular catheter, the differential diagnosis of shunt-related foreign body granuloma versus recurrent low-grade glioma was raised. Although chronic encapsulated intracerebral hematomas have been reported in the literature, the anatomical location of the lesion in the presented case was unique, with radiological and history findings also posing a peculiar diagnostic challenge. Chronic encapsulated intracerebral hematomas are benign entities that may also be found to involve deep and midline supra-tentorial structures usually not prone to spontaneous intraparenchymal hemorrhages. When symptomatic, surgical resection of the hematoma can be both diagnostic and curative.

## Introduction

Chronic encapsulated intracerebral hematoma (CEIH) is a well-known complication of spontaneous intraparenchymal hemorrhage (ICH) characterized by the slow enlargement of the hematoma due to recurrent micro-hemorrhages within its capsule [1]. The frequency of CEIH after spontaneous ICH was found to be 4.9% in a recent study where the presence of a radiological “layer sign” surrounding the hematoma (as a sign of recurrent bleeding) was found to be predictive of progressive hematoma expansion [2]. In patients with no clinical history of symptomatic spontaneous hemorrhage, vascular malformations, previous radiation therapy, or radiosurgery, or when other intracranial conditions are present (as it was in our patient), the diagnosis may be challenging. Midline structures, such as the corpus callosum and the septum pellucidum, are uncommon sites for spontaneous ICH and therefore not the expected anatomical location for the formation of an CEIH. We present a very rare case of chronic encapsulated hematoma involving the septum pellucidum and the foramen of Monro that by location, radiological appearance, and clinical history was mimicking a recurrent astrocytoma or a shunt-related foreign body granuloma. To the best of our knowledge, no other cases of CEIH involving the septum pellucidum and the foramen of Monro have been previously reported in the literature.

## Case presentation

**Figure 1:**
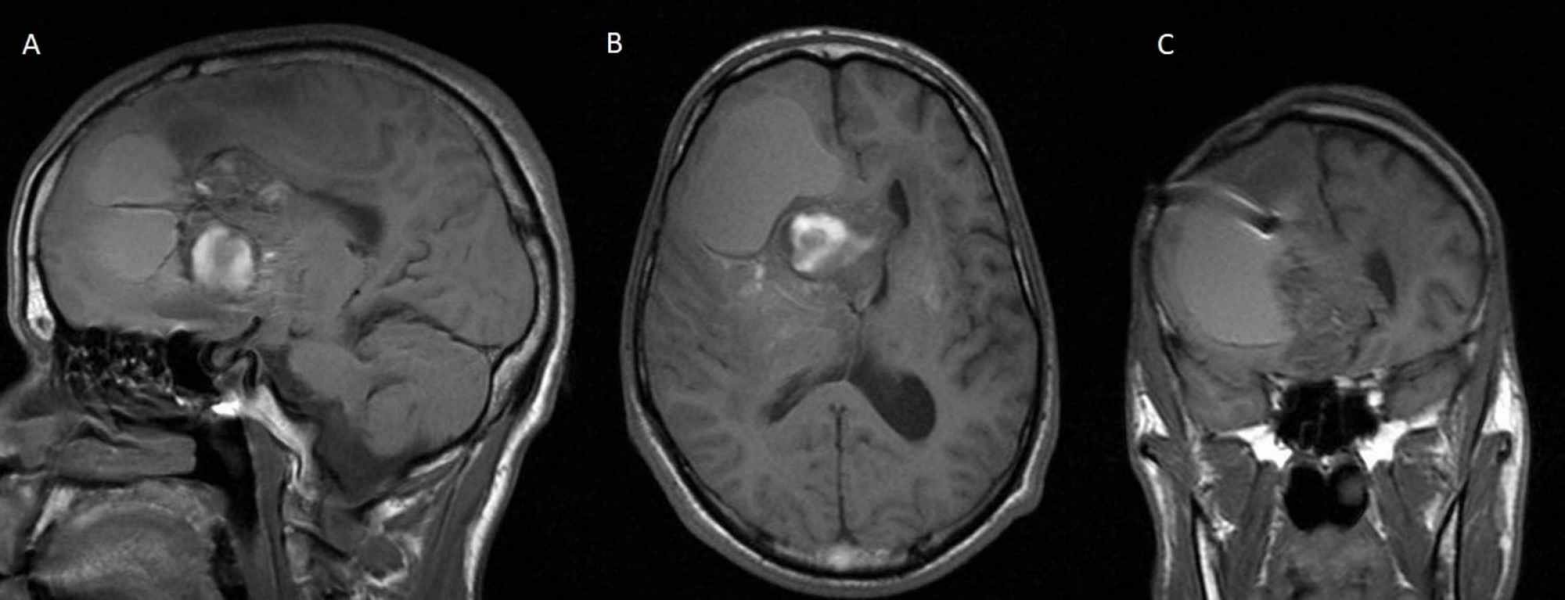
Preoperative sagittal (A), axial (B), and coronal (C) T1-weighted MRI without contrast, showing the solid cystic mass centered near the septum pellucidum. The shunt catheter is seen ending inside the solid mass on the coronal image (c).

**Figure 2:**
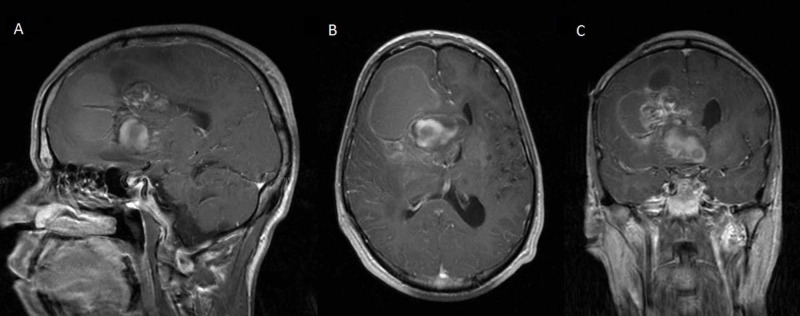
Preoperative sagittal (A), axial (B), and coronal (C) T1-weighted MRI with contrast, showing the solid cystic mass centered near the septum pellucidum. Only faint enhancement after gadolinium injection is seen within the solid mass.

A young adult presented to our evaluation after an accidental fall with head injury and brief loss of consciousness. He had a history of cognitive disability with short-term memory deficit since early childhood when he was operated for a resection of a hemispheric frontal juvenile pilocytic astrocytoma (JPA), as per family report. As a child, he also sustained a severe head injury in a motor vehicle accident (MVA) that led to a posttraumatic hydrocephalus (HCP) that was treated with the placement of a right frontal ventriculoperitoneal (VP) shunt. After a few years, the patient underwent a shunt revision for recurrent HCP where the extracranial portion of the old shunt was tied and disconnected and a new right parietal shunt was placed. The patient had no further follow-ups with the neurosurgery service, and his cognitive deficits remained stable, with stable mild gait ataxia/instability and occasional urinary urgency. At ER evaluation, a CT of the head and MRI of the brain revealed the presence of a heterogeneous solid mass involving the region of the right foramen of Monro and septum pellucidum with a large right frontal cystic component. The solid and deep mass had only minimal faint enhancement after gadolinium injection, and there were no peripheral enhancement or HCP. There was no restricted diffusion on DWI (diffusion-weighted imaging) and unenhanced T1-weighted images, but was some increased signal intensity consistent with small areas of hemorrhage. The mass was encasing the tip of a shunt catheter, whereas another ventricular catheter was ending within the right lateral ventricle (Figures 1, 2). These findings were consistent with the patient’s history of VP shunt placement and revision.

The patient had no history of recurrent headaches, loss of consciousness, and no progressive symptoms as per family members’ report. Aside from the MVA, no other recurrent head traumas were reported. In light of the clinical and radiological findings, a differential diagnosis comprehensive of foreign body granuloma and recurrent JPA was raised.

The patient (with the support of his family) elected to undergo a right frontal craniotomy for resection of the mass, decompression, and diagnosis, which was uneventful. He recovered well, his gait improved, and he was able to resume some work activities a few weeks after the surgery.

**Figure 3:**
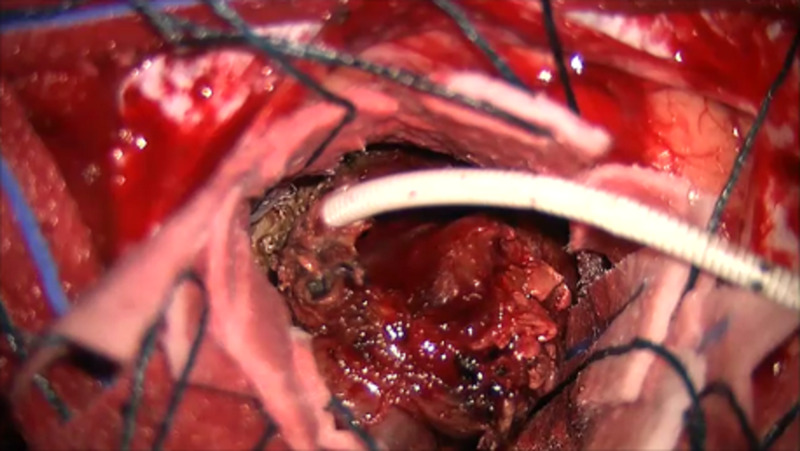
Intraoperative images of the shunt catheter surrounded by the chronic encapsulated hematoma.

**Figure 4:**
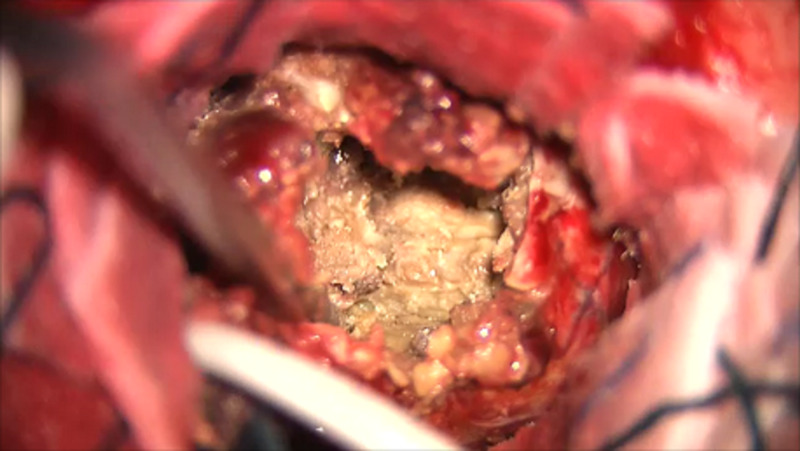
Gross appearance of the content of the encapsulated hematoma during surgical debulking.

At the time of the surgery and after decompressing the frontal cyst, a solid mass consistent with an organizing hematoma was found and removed from the surrounding gliotic parenchyma. There were no feeders to the mass, and an old frontal flanged, spring reinforced, Portnoy ventricular catheter was found at the center of the organizing hematoma (Figures 3, 4).

**Figure 5:**
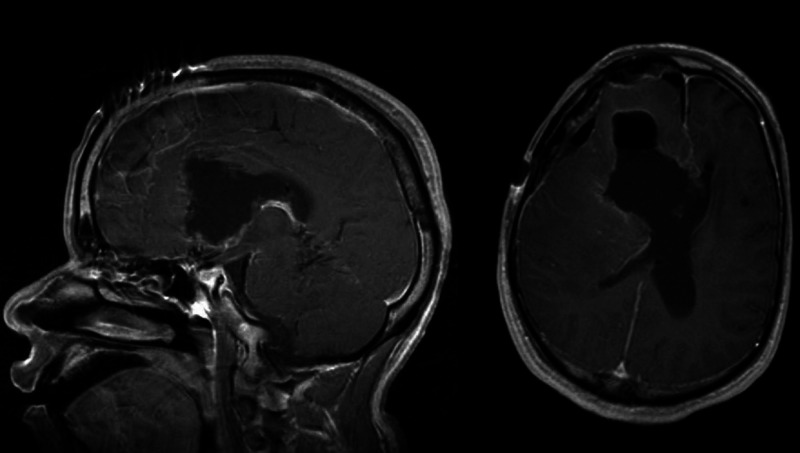
Postoperative sagittal and axial T1-weighted MRI with contrast, showing complete resection of the encapsulated hematoma and retained nonfunctioning shunt.

Postoperative MRI confirmed the complete resection of the mass, and there were no new postoperative deficits (Figure 5).

**Figure 6:**
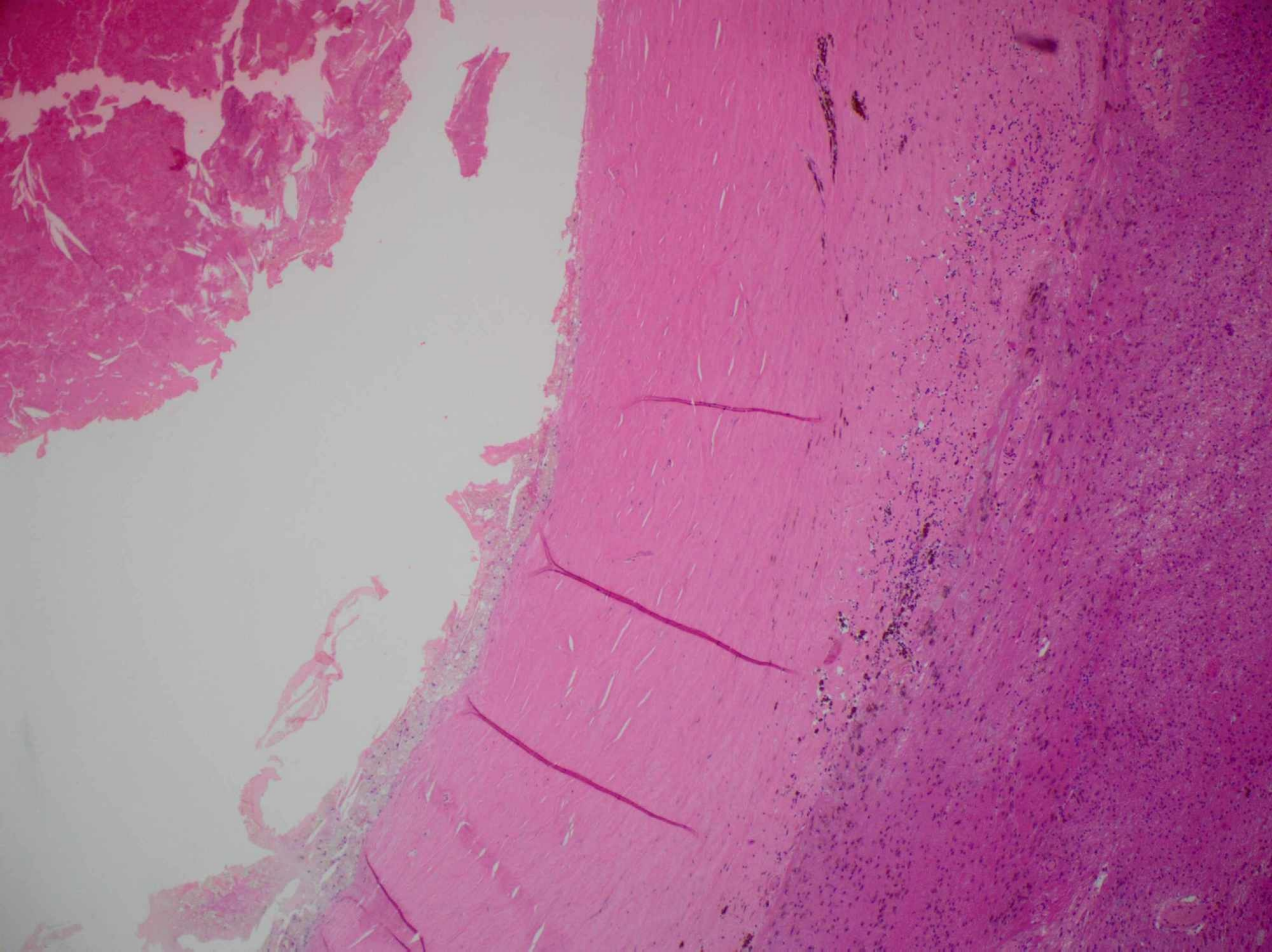
Histology image showing the hematoma capsule with hemosiderin and gliosis (H&E 4x).

**Figure 7:**
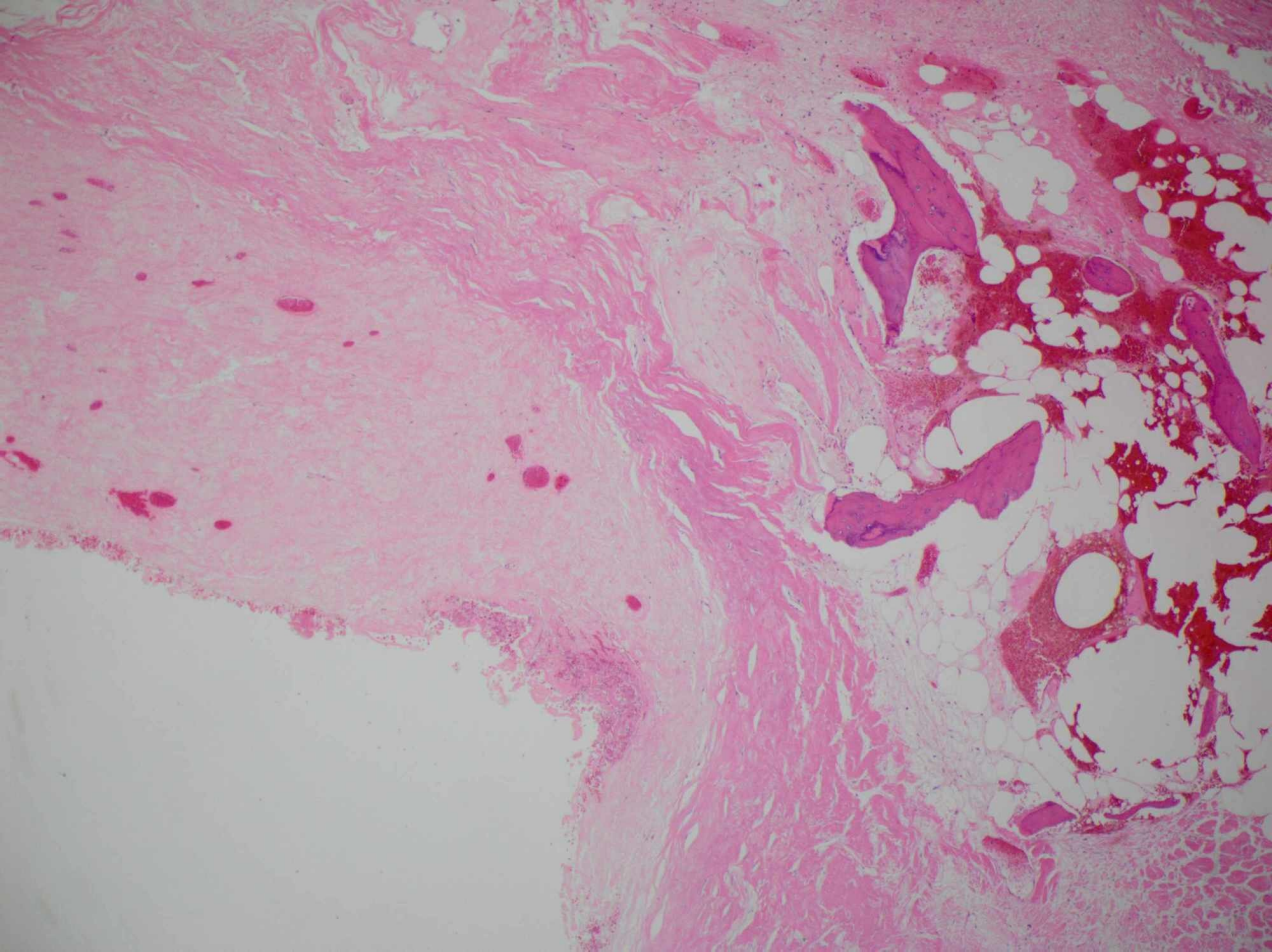
Histology image showing the hematoma fibrous capsule with osseous metaplasia (H&E 4x).

**Figure 8:**
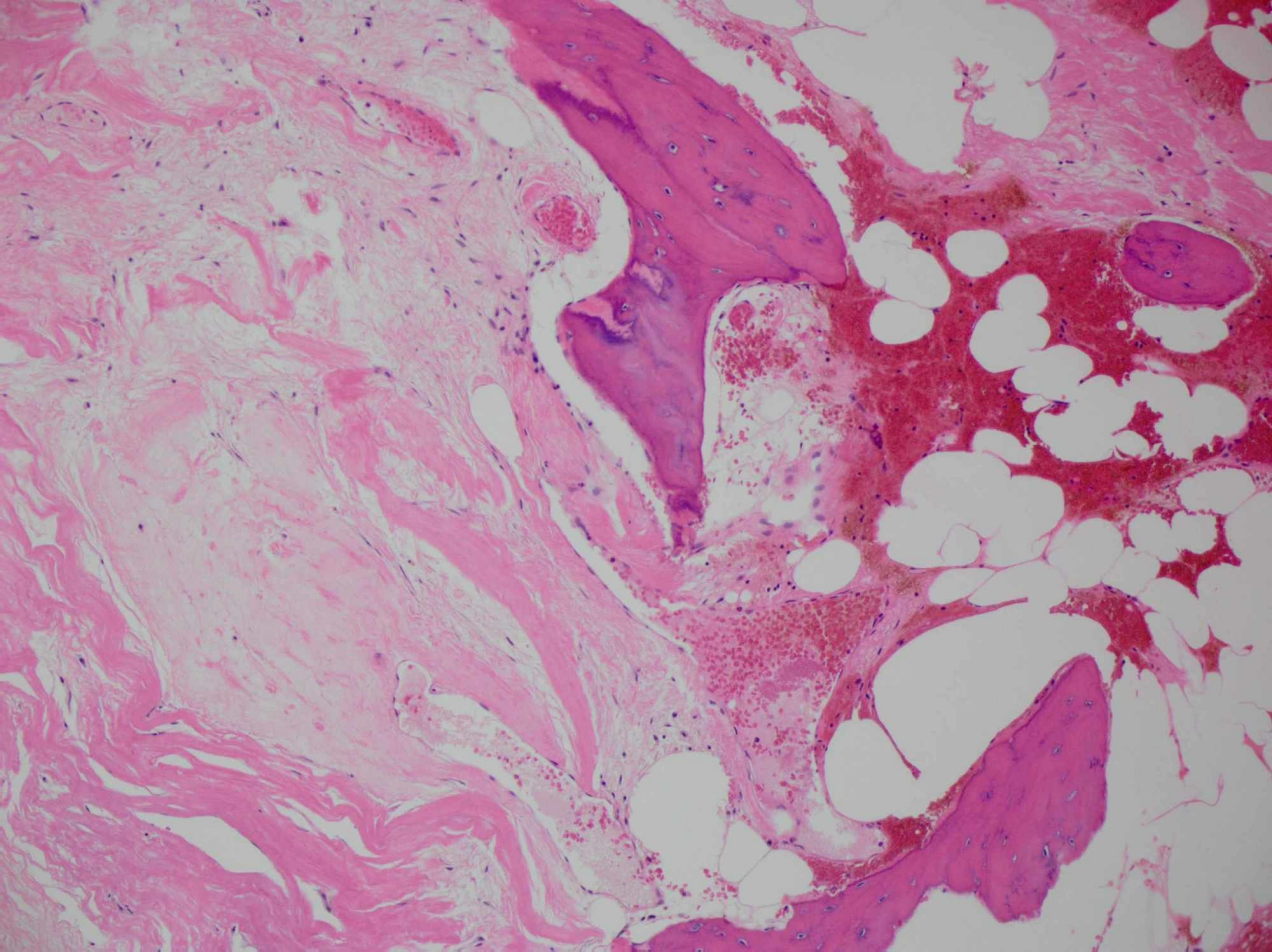
Histology image showing the hematoma fibrous capsule with osseous metaplasia (H&E 10x).

Pathology examination revealed a mass tightly adherent to the Portnoy shunt catheter. The lesion wall had multiple calcified foci with central hemorrhagic necrotic friable material. Histological sections reveal a dense fibrous hyalinized capsule with osseous metaplasia. Various stages of organizing hematoma were present within the mass. There were no foreign body giant cells or granulomas to suggest a foreign body granuloma. Surrounding this encapsulated hematoma, there was non-neoplastic brain tissue with reactive gliosis and hemosiderin (Figures 6-8). No evidence of residual or additional tumor was seen in the entirely sampled tissue.

The findings were consistent with the final diagnosis of CEIH with osseous metaplasia.

## Discussion

CEIH is a rare type of intracerebral hematoma first described by Hirsh et al. and characterized by the presence of a fibrotic capsule surrounding an organizing hematoma [3]. CEIH is a well-known complication of spontaneous ICH and its formation is thought to be secondary to recurrent episodes of micro-hemorrhages within its capsule, which lead to hematoma growth and the possible onset of slowly progressing neurological symptoms [1]. Radiological diagnosis with CT and MRI scans is not always straightforward, as multiple hemorrhages in different stages of evolution can render every case peculiar; therefore the use of thallium-201 single-photon emission CT has been suggested during the preoperative diagnosis [4]. CEIH formation has been described as a late sequela after stereotactic radiosurgery for arteriovenous malformations (AVMs) [5], with activation of vascular endothelial growth factor pathways that leads to hematoma capsule neovascularization, subsequent recurrent mural bleedings, and progressive hematoma enlargement [6].

CEIHs have been reported to involve the hemispheric convexity, the basal ganglia, and the cerebellum [7-9]. Although they are more commonly seen in adults [10], pediatric cases have also been described [11]. These hematomas can be clinically indolent and paucisymptomatic or present with slowly progressing neurological symptoms due to recurrent bleeding within the hematoma capsule. Some patients may also present with a history of epilepsy or intractable seizures [12]. On pathology examination, the hematomas appear well capsulated with the capsule made of an outer fibro-collagenous layer and an inner granulation layer with high concentrations of vascular endothelial growth factor [6]. Chronic encapsulated hematomas have been reported in association with vascular lesions, such as cavernous malformations and ruptured AVMs [13,14], and have been recognized as a late complication of radiosurgery treatment when the AVMs were not completely obliterated [6]. They may also mimic hemorrhagic primary and secondary brain tumors [15], and, in the presented case, the hematoma surrounding an old ventricular flanged catheter raised the suspicion for the presence of a shunt-related foreign body granuloma.

The case presented is original in that the chronic encapsulated hematoma developed years after a resection of a low-grade glioma (frontal JPA), was strictly associated with a first-generation retained nonfunctioning ventricular catheter, and demonstrated osseous metaplasia of the capsule. The anatomical location of the pathology was also very uncommon and not previously reported. The presence of immature bone formation (osseous metaplasia) within the hematoma capsule has been previously described in subdural, and rarely interdural, hematomas [16,17] and was a distinctive and rare feature on the specimen reported here.

The differential clinical and radiological diagnosis included recurrent low-grade tumor and foreign body granuloma. Pilocytic astrocytomas are low-grade gliomas most commonly seen in childhood and can be cured with complete surgical resection. In adults, they can present as adult pilocytic astrocytomas or reflect a recurrent tumor after an incomplete surgical resection at an early age [18]. Pilocytic astrocytoma demonstrates a myxoid background and numerous monomorphous bipolar cells, whose processes often radiate from prominent blood vessels. The brain tissue in this specimen did not demonstrate myxoid change or enough increased cellularity and vascularity for a diagnosis of recurrent pilocytic astrocytoma to be considered. Foreign body granulomas have been reported as a complication of CSF shunting procedures, both intracranially and in the spine, with the granuloma formation elicited as a reaction to the presence of the catheter material [19,20]. The old Portnoy flanged ventricular catheter was spring reinforced, therefore adding to the possibility of an allergic chronic reaction to the shunt placement. Granulomas around a foreign body consist of inflammatory cells infiltrate, with giant cells, lymphocytes, plasma cells, epithelioid cells, and perivascular cuffing. All these findings were absent in the presented case.

In our case, it is unclear if the initial ICH that led to the formation of the CEIH was related to an intraoperative bleeding in the region of the foramen of Monro/choroid plexus at the time of the shunt revision, was secondary to a minor trauma, or was possibly related to a rare hemorrhage within a residual JPA.

## Conclusions

CEIHs involving the septum pellucidum and the foramen of Monro are very rare, and, when associated with the presence of a ventricular shunt, may mimic the appearance of a shunt-related foreign body granuloma. Although radiological preoperative examinations can facilitate narrowing the differential diagnosis, surgical resection of the encapsulated hematoma is both diagnostic and curative.
